# The Good Samaritan Parable Revisited: A Survey During the COVID-19 Pandemic

**DOI:** 10.3389/fpsyg.2022.776986

**Published:** 2022-04-13

**Authors:** Yong Lu

**Affiliations:** Institute of Psychology, Faculty of Christian Philosophy, Cardinal Stefan Wyszyński University in Warsaw, Warsaw, Poland

**Keywords:** COVID-19, empathy, helping behavior, prosocial behavior, regulatory focus theory

## Abstract

From an integrative approach of parable interpretation that combines ethical, evolutionary, historical, and psychological perspectives, the current research empirically examined the purely theorized assumption elucidating the behaviors of the priest, Levite, and Samaritan in the good Samaritan parable (Luke 10:25-37) by the regulatory focus theory. In one experiment conducted during the COVID-19 outbreak, 93 Polish participants were randomly assigned to a simulated vignette of the good Samaritan parable where either the prevention or promotion regulatory focus was manipulated. The results confirmed a certain favorable tendency to offer quasi-realistic help in both the regulatory focus conditions. The finding highlights a dynamic association in goal pursuit motivation and prosocial behavior in a pandemic context regarding the good Samaritan parable. The current study is among rare empirical research which reflects a challenge people respond to offer help in simulated scenarios as original as the good Samaritan parable.

## 1. Introduction

The parable of the compassionate Samaritan (Luke 10:30-37) has been among the most famous narrative portrayals that were exemplified by Jesus. In the parable, Jesus is abruptly interrupted by an expert in the law who intends to test the Lord (Luke 10:25). The expert asks, “Who is my neighbor?” (Luke 10:29b, *The Bible*, New International version and so subsequently). As the majority of rabbinic parables functions as exegeses of the scriptural text or narrative (Jeremias, 2003, pp. 112-113), Jesus puts forward the parable in order to elucidate the greatest commandment—“Love your neighbor as yourself” (Leviticus 19:18b; Luke 10:27b)—and a most proclaimed Confucius' and Hellenistic “golden rule”—“What you do not want [sic] done to yourself, do not do to others” (refer to *Analects* 15.24; Luke 6:31; cf., Lu, 2020). In the parable, Jesus narrates that while a priest and a Levite journey away on the Jericho road from Jerusalem, they sequentially pass by and neglect an injured man. Nonetheless, to somewhat the audience's surprise, an ignominious Samaritan, who performs as an example of one who loves his neighbor, comes to help the victim by bringing him to a sheltered place (cf., Luke 9:58b) and by instructing the innkeeper to spare no expense in his treatment (cf,. Luke 2:7b; refer to Longenecker, 2009 for a characteristic interpretation on the innkeeper). However, it should be noted that the injured man is seemingly beaten unconscious by the robbers who also steal his clothes. As a result, the audience, being Jewish, can not identify, without his dialect and dress, what clan the injured man is belonged to and how much he is involved in his religion (cf., Green, 1997, p. 429). The paradigm of the good Samaritan parable serves to demonstrate that the demand to love God has to be complemented by what God demands of the love of the neighbor. Further syntheses of the parable can be found in 2 Chronicles 28:15 and Deuteronomy 10:18-19, and further hermeneutic and hypertextual exegeses appear in Adamczewski (2010, pp. 319-322) and Proctor (2019).

In Temple times, priests and Levites were assumed to be different from each other with regard to ritual cleanness and uncleanness (Leviticus 10:10). More precisely, they were urged to maintain the natural state and to keep away from impurity, both of which belong, of any kind, to the disintegration of the body. As one of the central religious concerns and awareness in Judaism, impurity is a specifically nonstandard status to the extent to which a person loses his or her status of ritual purity because of inappropriate bodily processes or sins (Wenham, 1979, p. 23). Importantly, according to the Bible (cf., Leviticus 15 and Deuteronomy 23:11-12), the impurity can be imparted by certain sources of pollution, such as direct or indirect contact with a dead human body, blood (menstruation), sweat, scale diseases (i.e., leprosy), or any discharges from privy parts or sexual organs (e.g., excrement or urine). Particularly, the impurity can be even transmitted through the air from corpse contamination in certain extreme circumstances (cf., Exodus 19:14-22; Douglas, 1966, p. 51; Fröhlich, 2010, pp. 2-3). Nonetheless, human impurity is not regarded as a sin but simply a natural phenomenon that is often related to the natural functioning of the body. However, if priests and Levites defiled themselves (e.g., a contact with a dead body), they could neither enter the Temple's territory (courtyards) nor receive, give, and consume tithes. Specifically, even though priests had a responsibility to bury abandoned corpses, the defilement contaminated by corpses was still seen as the strongest impurity (cf., Salo, 1991, p.110; for ethical perspectives on priests and Levites, refer to Clark, 2014). Qumran records that death pollution makes impure the entire inner space of the house, i.e., whatever and whoever in the house, and they shall maintain unclean for 7 days (e.g., 11Q 19 XLIX.10; for general reviews on the system of ritual purity and impurity in Judaism, refer to Wright, 1992; Woolf, 2015).

According to the Bible, the Samaritans were descendants of Assyrians who settled in the former kingdoms of the land of Northern Israel in the Sargon time (cf., 2 Chronicles 28, Ezra, 2 Kings 17, Nehemiah; refer to also Fensham, 1982, p. 67; Frey et al., 2012). A large number of research regarding the Samaritan Pentateuch, papyri, inscriptions, archaeological discoveries, and others indicate that the Samaritans assembled as a small-sized communal group and resided in certain, locally bounded places near the temple on Mount Gerizim (refer to Fensham, 1982, p. 18). Moreover, the Samaritans preserved their seemingly self-rooted religious kinship systems, as mostly represented by a surrogate form of worshiping venerated yhwh, the God of Israel (cf., Anderson and Giles, 2002, pp. 24-34 and Pummer, 2010 for a brief introduction). According to Tanaitic sources, “The ways of the Cuthites are sometimes like idolaters, sometimes like Jews. Most of them are like Jews” (Tractate Cuthim 1,1), the Cuthites/Samaritans are classified neither as Jews nor as idol worshipers. Besides, the Second Temple Jews syncretized that the Samaritans were antitheses between Israelites and pagans with regard to their collective identities, politics, and religious interests (Kartveit, 2009; cf., Matthew 10:5; John 4:9, 8:48, 9:51-56; for conjectural employments of the Samaritans and the historicity and socialization of relevance to the Second Temple period, refer to Knowles, 2004). Nevertheless, the Samaritans remained faithful servants of Israel's God. More recently, Chalmers (2020) argued against the conventionally scholarly exclusion of the Samaritans in first-century Palestine from Israelites—they were nevertheless understood by Jews as enmities; rather, the status of the Samaritans was presumably situated as an interactive inclusion of Samaritan Israelites rather than non-Israelite “others.” Compared with Jews, due to a lack of knowledge of the ritual precept, the Samaritans were occasionally suspicious of their strictness of abiding by the legal system of impurity and purity (Amit, 2010, p. 263). In spite of the fact that there existed many rebuttals in late antiquity against the Samaritans by the Jewish tradition (refer to Schreiber, 2014, ch. 2), the Gospels show sympathetic perspectives on the ostensibly “alien” Samaritans (e.g., Luke 10:25-37, 17:16; John 4:39-42).

The present article aimed to empirically rephrase Lu's (2017a) theoretical study on the explanation of the good Samaritan parable by the regulatory focus theory. In the next section, we delineate this explanation from an integrative approach that combines ethical, evolutionary, historical, and psychological perspectives. Accordingly, we formulate a hypothesis for this novel argument featuring the parable in a more contemporary context. Then, we examined this hypothesis in a behavioral-judgmental experiment.

## 2. A Social, Evolutionary, and Behavioral Perspective on the Good Samaritan Parable

The narrative of the good Samaritan parable has been rated by the Jesus Seminar with 60% to be authentic and 29% to be probably authentic (Jones, 1999, p. 294). While many hermeneutical exegeses, humanitarian perceptions, socially justified law courts, and other fields have addressed it (for recent discussions, refer to Zimmermann, 2015; Rule, 2017; Zylla, 2017), only very limited social-psychological studies have attempted to duplicate a verisimilar situation in order to authentically examine the original parable's implications in the contemporary context. In fact, there appears to be only one relevant study so far by Darley and Batson (1973), who conducted a simulated good Samaritan field experiment in a between-subject design for male seminary participants who encountered a real “victim.” The results showed that the experimental group who was primed to consider religious and ethical thoughts (i.e., a talk on the good Samaritan parable) was not more inclined to call for a helping response than the control group who was primed to other topics (e.g., a talk on the jobs instead).

Nevertheless, the research from contemporary psychological studies may provide insights into our understanding of the narrative of the good Samaritan parable. Recently, Lu (2017a) introduced the regulatory focus theory, which posits that a person intends to pursue a behavior in a way that maintains the person's own orient standpoints and desires (Higgins, 2012), as a theoretical approach to interpreting the behaviors of the priest, Levite, and Samaritan in the parable. According to the theory, there coexist two distinct self-regulatory focuses: *prevention* and *promotion* (Higgins et al., 1994; Higgins, 2008; Hodis, 2017). Individuals whose self-regulation has a prevention focus are inclined to perform a defensive strategy, which may lead to a high vulnerability to pessimistic circumstances, such as the prevention of errors and losses and the fulfillment of responsibilities. In contrast, when a promotion focus is dominated, individuals are likely to prefer an enthusiastic strategy, which may lead to a particular sensitivity to positive information, such as the pursuit of gains and aspiration toward ideals and hedonic pleasure (e.g., Crowe and Higgins, 1997; Uskul et al., 2009; Gino and Margolis, 2011). Past research has demonstrated the impact of regulatory focuses (e.g., prevention vs. promotion) on such as (un)ethical behaviors (e.g., Gino and Margolis, 2011) and decision-making strategies (e.g., Lu and Nieznański, 2017).

According to Lu's (2017a) purely theoretical conjecture, the priest and Levite in the good Samaritan parable were largely affected by their ritual restrictions, whereas the Samaritan was much less likely affected by his religious constraints as to which he must strictly abide by the similar laws of injunction against contact with the dead. Accordingly, the priest and Levite considered their defilement, specifically when it can be caused by touching a corpse, as their vital distress of humiliation. In contrast, it is argued that the Samaritan would regard his possible defilement as less serious suffering in consideration of his impertinent involvement relating to any religious services. Furthermore, the priest and Levite pursued a sturdy prevention focus of their self-regulation goals by taking into consideration any potential threats against safety, security, and vigilance. Thus, they neglected the victim in order to avoid a potentially risky menace of defilement. The Samaritan, however, had no goals to sustain the law of ritual cleanness, so his self-regulation goals were much less affected by the prevention focus. This resulted in the matter of fact that his empathy could override the risk of defilement and then could choose to help the victim. Nevertheless, the limitations of this interpretation were also addressed in that, in short, it applies contemporary psychological perspectives to the explication of a particular historical pericope. A similar elucidation was also done by Lu (2017b) who proposed a novel application of the false memory theory on the exegesis of Peter's denials of Jesus, but Howes (2017) criticized such attempts due to the conscious removal of the pre-Easter context when explicating the parables.

From an evolutionary point of view, the self-regulatory focuses of contemporary priests and Levites have changed tremendously, compared with the priest and Levite in the parable who were restricted by the ritual purity law, ethnically remote away from us, in specific areas of the Mediterranean, long ago. Nowadays in most areas, priests and Levites have not been necessary to daily abide by the ritual law, apart from celebrating the Eucharist or reading the Torah in the synagogue. Therefore, their occupational obligations have evolutionarily transformed from obeying the Jewish law into, e.g., showing themselves as good shepherds and practicing the idea of divine mercy toward people who need help. Furthermore, after hundreds of years of preaching the Gospels, both religious belief and helping behavior are all correlated with Christian ethical virtues such as “having love and compassion for one's fellow man” and “being a good Samaritan” (Cline and Richards, 1965). This assumes a positive association between religiosity and prosocial tendency toward outgroups (e.g., Galen, 2012; Batara et al., 2016). Although contemporary research has shown that religious prosociality has been merely applied in certain circumstances (Norenzayan and Shariff, 2008), the explicit expression of the notion of agape or benevolence in the good Samaritan parable per se becomes a successful moderator to attenuate discrimination in certain instances (e.g., Johnson et al., 2015). Besides, the evidence from social identity theory showed that the application of the good Samaritan parable can directly reduce intergroup conflicts (Esler, 2000).

From a behavioral decision-making perspective, it is convincing to argue that the priest, Levite, and Samaritan in the parable took actions of either neglecting or helping the victim in a “completely” uncertain situation, where they could perceive the set of possible outcomes (e.g., defilement, delay/punctuality, mercy) for each action, but had no information about the probabilities of these outcomes. Consequently, each of the actions was related to an undetermined expected value represented by the set of possible outcomes corresponding to that action. Moreover, the decisions made by the priest, Levite, and Samaritan were unavoidably influenced by their underlying motivations and perceptions. Taken together, to summarize the variables suggested as affecting neglecting or helping behavior by the parable applied in contemporary society, the situational variables include the contents of one's fulfillment of obligations by priming prevention and non-regulatory focuses. The dispositional variables seem to involve types of religiosity. These variables suggest the following hypothesis:

**Hypothesis**. *When presumably encountering a situation possibly calling for an aiding response, individuals who are primed in a promotion regulatory focus condition by emphasizing on achieving gains are more likely to offer help than individuals who are primed in a prevention regulatory focus condition by emphasizing avoiding losses*.

## 3. Methods

### 3.1. Participants

A total of 93 Polish participants, who were randomly assigned to the two regulatory focus conditions, participated in the experiment. The female percentage was 54.84%, and the mean age was 29.3 years (*SD* = 9.3). They were invited through emails *via* social media. The current research was one of three online experiments in which each participant received 50 PLN (Polish currency Złoty) (1 PLN worth approximately €0.24 at the time of the experiment) in total as compensation for their participation. The payments were made as online shopping cards from a Polish commercial retailer.

### 3.2. Design and Materials

We presented to the participants a quasi-realistic vignette of the good Samaritan parable, asking about the likelihood of the neglect or help behavior by considering three consequences: Defilement, delay/punctuality, and mercy. The questionnaires were initially written in English and then were translated into Polish. The scenarios were back-translated to English in order to check that all the translated versions had the same contents. Half of the participants (*n* = 45) read the following scenario which evokes the prevention regulatory focus. Then, the participants responded to the question “You will neglect the victim” by indicating the extent to which they agreed with this statement (from 1 = *Strongly disagree* to 5 = *Strongly agree*).

Please imagine that while one day you are proceeding to take an exam at your university, you come across a half-dead victim with leprosy, who is left lying down by an alley. If you choose to neglect the victim, there is a 50% probability that you will be contagiously defiled by the skin disease, a 0% probability that you will be late to the exam, and a 100% probability that you will feel a lack of mercy (refer to Table 1). Please note that these three cues, i.e., defilement, delay, and mercy, are independent of each other.

**Table 1:** The probabilities resulted from neglecting the half-dead stranger with leprosy^a^.

**Defilement**	**Delay**	**Mercy**
− (50%)	+ (0%)	− (100%)

a *On a scale from 0% to 100%, ranging from - (loss) to + (gain), with probabilities in bracket (50% = chance level)*.

The other half of the participants (*n* = 48) read the following scenario which evokes the promotion regulatory focus. Then, the participants responded to the question “You will help the victim” by indicating the extent to which they agreed with this statement (from 1 = *Strongly disagree* to 5 = *Strongly agree*).

Please imagine that while one day you are proceeding to take an exam at your university, you come across a half-dead victim with leprosy, who is left lying down by an alley. If you choose to help the victim, there is a 50% probability that you will be contagiously defiled by the skin disease, a 0% probability that you will be punctual to the exam, and a 100% probability that you will gain a compliment of mercy (refer to Table 2). Please note that these three cues, i.e., defilement, punctuality, and mercy, are independent of each other.

**Table 2:** The probabilities resulted from helping the half-dead stranger with leprosy^a^.

**Defilement**	**Punctuality**	**Mercy**
+ (50%)	− (0%)	+ (100%)

a *On a scale from 0% to 100%, ranging from - (loss) to + (gain), with probabilities in bracket (50% = chance level)*.

The probabilities of the consequences of defilement, delay/punctuality, and mercy that were presumably resulted from the neglect or help behavior were displayed using the format shown in Tables 1, 2, respectively. We constructed the average cue validations to be the same; therefore, the manipulations of the two regulatory focuses may be not influenced if the participants used normal criteria such as mean validations. Furthermore, we manipulated the scenario as a binary, weak-dominant three-attribute alternative choice problem, in which the two behaviors contain the quantity-same, albeit direction-opposite, cue validations for the three consequences, respectively (i.e., defilement: − 50 vs. + 50%; delay: + 0 vs. − 0%; mercy: − 100 vs. + 100%); therefore, the manipulations of the two regulatory focuses may also be not influenced if the participants used aggregation heuristics such as the equate-to-differentiate rule (Lu, 2016) or the majority rule (Lu and Nieznański, 2017).

In order to check the effectiveness of the manipulations of the two regulatory focuses, we asked all the participants to answer the following questions: While you were reading about the scenario and question, please describe the extent to which (1) you thought about safety; (2) you thought about hope; (3) you thought about responsibility/obligation; (4) you thought about the accomplishment; (5) you thought about the avoidance of any losses; and (6) you thought about the pursuit of any gains. They responded on a 7-point Likert scale by indicating the extent to which they agreed with each statement (from 1 = *strongly disagree* to 7 = *Strongly agree*). The first, third, and fifth items were summed and averaged to form a prevention index, and the remaining three items were summed and averaged to form a promotion index.

### 3.3. Procedure

The experiment was conducted online during the COVID-19 outbreak, from December 2020 to February 2021. The participants received *via* email one leaflet containing the questions in PDF format (refer to Appendix A,B), and they answered individually at their own self-pace.

## 4. Results and Discussion

**Figure 1:**
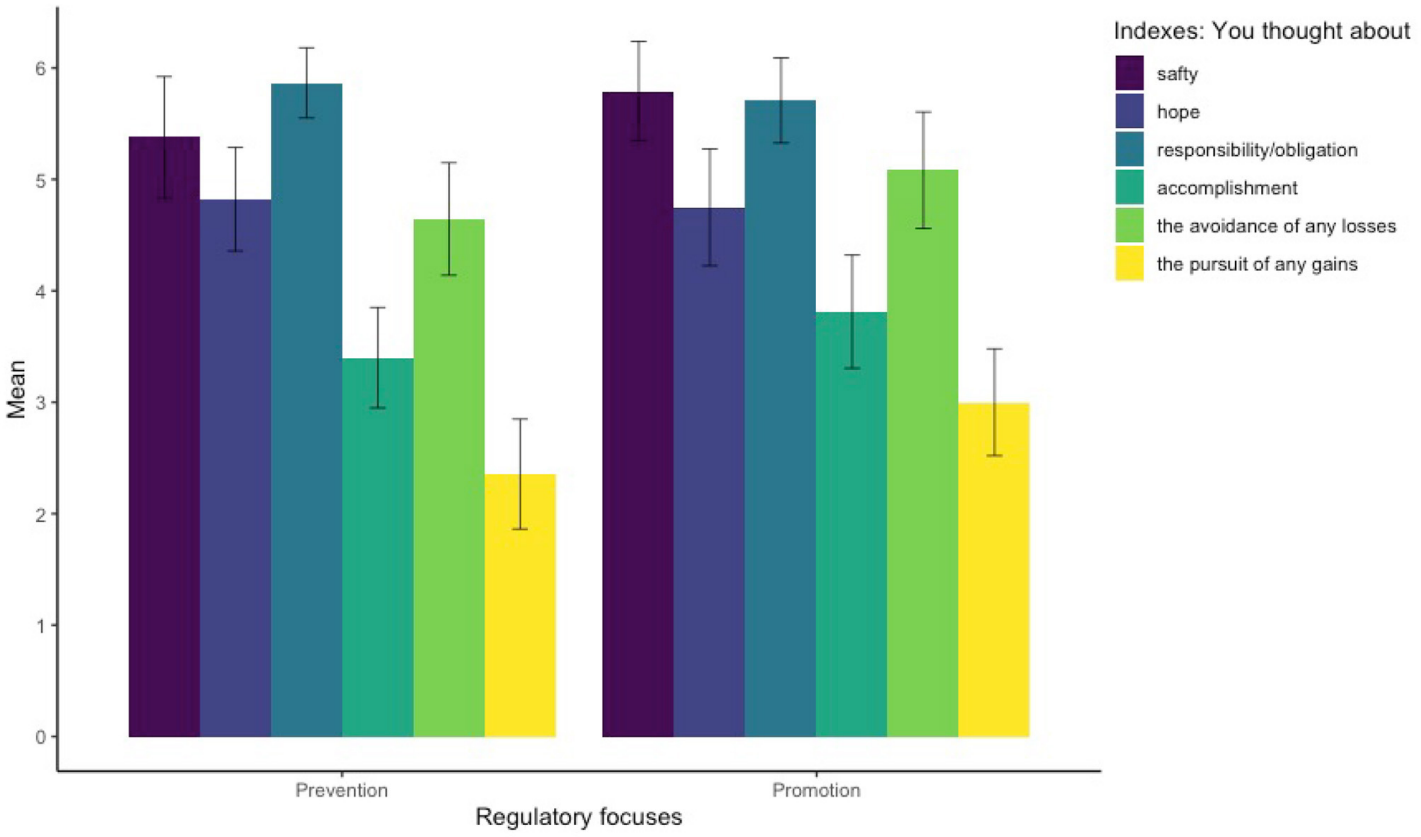
Prevention and promotion indexes. On a 7-point Likert scale (from 1 = *strongly disagree* to 7 = *Strongly agree*). Error bars are the ±2 standard error of the mean.

Cronbach's α coefficients for the prevention and promotion indexes were .58 and .54, respectively, suggesting that the participants' responses reached relatively acceptable internal consistency. During the COVID-19 outbreak, people have arisen considerable concerns about, generally speaking, safety, responsibility/obligation, and the avoidance of losses that are in line with the scope of the prevention regulatory focus. Compared with the non-outbreak period, for instance, the extent degree of anxiety about safety in the Polish population has become significantly higher during the outbreak period (Debowska et al., 2020; Gawrych et al., 2021; Malesza and Kaczmarek, 2021). Our results confirmed this pattern. On the one hand, the participants in the prevention condition thought more about safety, responsibility/obligation, and the avoidance of any losses (*M* = 5.30, *SD* = 1.63) than about hope, accomplishment, and the pursuit of any gains (*M* = 3.53, *SD* = 1.86), *t*(44) = 8.31, *p* < .001, *d* = 0.76. On the other hand, however, the participants in the promotion condition also thought more about safety, responsibility/obligation, and the avoidance of any losses (*M* = 5.53, *SD* = 1.59) than about hope, accomplishment, and the pursuit of any gains (*M* = 3.85, *SD* = 1.88), *t*(47) = 8.16, *p* < .001, *d* = 0.74. These results provided evidence that the manipulation primed the prevention regulatory focus effectively but, given the reality of the COVID-19 pandemic, not the promotion regulatory focus (cf., Figure 1).

**Figure 2:**
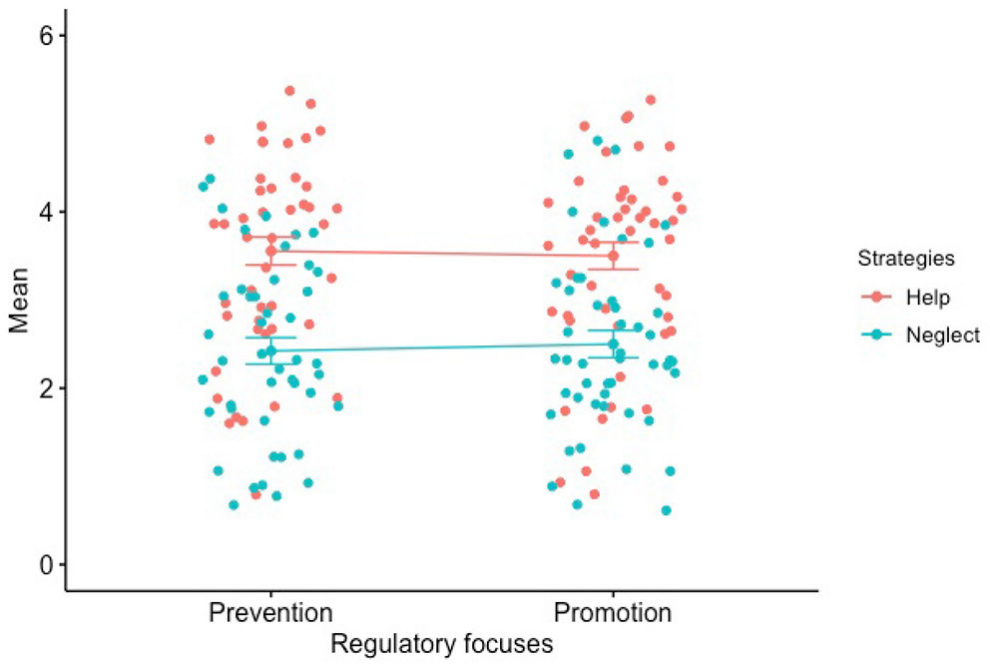
Neglect vs. help strategy. On a 5-point Likert scale (from 1 = *strongly disagree* to 5 = *Strongly agree*). Error bars are the ±1 standard error of the mean.

In the prevention condition, the mean rating that the participants were supposed to neglect the victim was 2.42 on the 5-point scale (*SD* = 1.01), i.e., a mean rating of 3.58 that the participants were supposed to help the victim. In the promotion condition, the mean rating that the participants were supposed to help the victim was 3.50 (*SD* = 1.07). The difference of the help strategy between the two conditions indicated a lack of significant level, *t*(91) = 0.36, *p* = 0.720, *d* = 0.05. It is argued that although the good Samaritan parable might serve as a COVID-19-related lesson in teaching helping someone in need (Chamburuka and Gusha, 2020), our results suggest only a certain favorable tendency to offer help, no matter what regulatory focus conditions individuals are situated (refer to Figure 2).

## Data Availability Statement

The datasets presented in this study can be found at the Open Science Framework website, at https://osf.io/de2gj. The data include all measures and conditions. No data are excluded from the analysis.

## Ethics Statement

The studies involving human participants were reviewed and approved by the local Institute Ethical Board of Cardinal Stefan Wyszyński University in Warsaw for Scientific Research (Evidence #: 15/2021). The participants provided their written informed consent to participate in this study.

## Author Contributions

YL designed the study, run the experiment, analyzed the results, and wrote the manuscript.

## Funding

This experiment was supported by Statutory Fund DEC-IP-6/20 from Cardinal Stefan Wyszyński University in Warsaw, Poland.

## Conflict of Interest

The author declares that the research was conducted in the absence of any commercial or financial relationships that could be construed as a potential conflict of interest.

## Publisher's Note

All claims expressed in this article are solely those of the authors and do not necessarily represent those of their affiliated organizations, or those of the publisher, the editors and the reviewers. Any product that may be evaluated in this article, or claim that may be made by its manufacturer, is not guaranteed or endorsed by the publisher.
