# Supplementary material for: The Good Samaritan Parable Revisited: A Survey During the COVID-19 Pandemic
Source: Front Psychol. 2022 Apr 13;13:776986. doi: 10.3389/fpsyg.2022.776986 (PMC9043862; doi:10.3389/fpsyg.2022.776986)
Supplement: Supplementary file 1 [file Data_Sheet_1.pdf]

Age:

Gender: Man ☐ Female ☐

Please imagine that while one day you are proceeding to take an exam in your university, you come across a half-dead victim with leprosy, who is left lying down by an alley. If you choose to neglect the victim, there is a 50% probability that you will be contagiously defiled by the skin disease, a 0% probability that you will be late to the exam, and a 100% probability that you will feel a lack of mercy (see Table 1). Please note that these three cues, that is, defilement, delay, and mercy, are independent of each other.

Table 1: The probabilities resulted from neglecting the half-dead stranger with leprosy[a]

Defilement	Delay	Mercy
- (50%)	+ (0%)	- (100%)

[a] On a scale from 0% to 100%, ranging from - (*loss*) to + (*gain*), with probabilities in bracket (50% = chance level).

You will neglect the victim:

While you were reading the scenario and the question, please describe the extent to which:

1. You thought about safty:
2. You thought about hope:
3. You thought about responsibility/obligation:
4. You thought about accomplishment:
5. You thought about avoiding any losses:
6. You thought about pursuiting any gains:

Age:

Gender: Man ☐ Female ☐

Please imagine that while one day you are proceeding to take an exam in your university, you come across a half-dead victim with leprosy, who is left lying down by an alley. If you choose to help the victim, there is a 50% probability that you will be contagiously defiled by the skin disease, a 0% probability that you will be punctual to the exam, and a 100% probability that you will gain a compliment of mercy (see Table 2). Please note that these three cues, that is, defilement, punctuality, and mercy, are independent of each other.

Table 2: The probabilities resulted from helping the half-dead stranger with leprosy[a]

Defilement	Punctuality	Mercy
+ (50%)	- (0%)	+ (100%)

[a] On a scale from 0% to 100%, ranging from - (*loss*) to + (*gain*), with probabilities in bracket (50% = chance level).

You will help the victim:

While you were reading the scenario and the question, please describe the extent to which:

1. You thought about hope:
2. You thought about safty:
3. You thought about accomplishment:
4. You thought about responsibility/obligation:
5. You thought about pursuiting any gains:
6. You thought about avoiding any losses:
